# Sequence- vs. chip-assisted genomic selection: accurate biological information is advised

**DOI:** 10.1186/s12711-015-0117-5

**Published:** 2015-05-09

**Authors:** Miguel Pérez-Enciso, Juan C Rincón, Andrés Legarra

**Affiliations:** Centre for Research in Agricultural Genomics (CRAG), CSIC-IRTA-UAB-UB Consortium, 08193 Bellaterra Barcelona, Spain; Departament de Ciència Animal i dels Aliments, Universitat Autònoma de Barcelona, 08193 Bellaterra Barcelona, Spain; Institut Català de Recerca i Estudis Avançats (ICREA), Carrer de Lluís Companys 23, Barcelona, 08010 Spain; Universidad Nacional de Colombia, Sede Medellín, Facultad de Ciencias Agrarias, Departamento de producción Animal, Medellín, Colombia; INRA, UMR 1388 GENPHYSE, Génétique, Physiologie et Systèmes d’Elevage, Castanet-Tolosan, 31326 France

## Abstract

**Background:**

The development of next-generation sequencing technologies (NGS) has made the use of whole-genome sequence data for routine genetic evaluations possible, which has triggered a considerable interest in animal and plant breeding fields. Here, we investigated whether complete or partial sequence data can improve upon existing SNP (single nucleotide polymorphism) array-based selection strategies by simulation using a mixed coalescence - gene-dropping approach.

**Results:**

We simulated 20 or 100 causal mutations (quantitative trait nucleotides, QTN) within 65 predefined ‘gene’ regions, each 10 kb long, within a genome composed of ten 3-Mb chromosomes. We compared prediction accuracy by cross-validation using a medium-density chip (7.5 k SNPs), a high-density (HD, 17 k) and sequence data (335 k). Genetic evaluation was based on a GBLUP method. The simulations showed: (1) a law of diminishing returns with increasing number of SNPs; (2) a modest effect of SNP ascertainment bias in arrays; (3) a small advantage of using whole-genome sequence data vs. HD arrays i.e. ~4%; (4) a minor effect of NGS errors except when imputation error rates are high (≥20%); and (5) if QTN were known, prediction accuracy approached 1. Since this is obviously unrealistic, we explored milder assumptions. We showed that, if all SNPs within causal genes were included in the prediction model, accuracy could also dramatically increase by ~40%. However, this criterion was highly sensitive to either misspecification (including wrong genes) or to the use of an incomplete gene list; in these cases, accuracy fell rapidly towards that reached when all SNPs from sequence data were blindly included in the model.

**Conclusions:**

Our study shows that, unless an accurate prior estimate on the functionality of SNPs can be included in the predictor, there is a law of diminishing returns with increasing SNP density. As a result, use of whole-genome sequence data may not result in a highly increased selection response over high-density genotyping.

## Background

The prediction of genetic merit, that is, the average phenotypic value of an infinite number of descendants from a given individual, has been the subject of active investigations in quantitative genetics for decades. Due to the rapid decrease in genotyping prices and to the development of new statistical methodologies, genomic selection is becoming the standard procedure for genetic evaluations in most relevant animal and plant species. Currently, there is ample consensus that genomic selection can be more efficient than traditional methods based only on pedigree information and phenotypes (best linear unbiased prediction, BLUP) and that this improvement more than offsets genotyping costs in many commercial breeding schemes, primarily for dairy cattle [1]. It is estimated that genetic progress in milk yield increased by roughly 50% in US Holsteins from 2006 to 2012 compared to the period from 2000 to 2006, coinciding with the implementation of genomic selection in the industry [2]. Initially, genomic selection was intended to use genotypes obtained from commercial single nucleotide polymorphism (SNP) arrays. However, more recently, the development of next-generation sequencing technologies (NGS) has made it feasible, or at least conceivable, that whole-genome sequence data could be obtained and used for routine genetic evaluations.

As a result, there is currently considerable interest in the animal and plant breeding fields on the use of high-throughput sequencing technologies for genomic selection [3,4]. Compared to SNP arrays, two main advantages of having whole-genome sequence data have been advocated: (1) the possibility of identifying causal mutations and (2) an increase in prediction stability that relaxes the need of updating the predictor every (few) generations [3]. This advantage is greater when rare variants explain a substantial part of the genetic variance [5]. Nevertheless, increasing the marker density of commercial chips has not increased accuracy of genomic predictions [6], and one explanation is that these chips do not capture rare allele variants. In addition, SNPs in arrays are a biased sample of all the SNPs that segregate in the population of interest (an ascertainment bias exists), whereas direct sequencing does not suffer, in principle, from this drawback.

Although sequencing prices are rapidly decreasing, routine analyses of NGS data from a massive number of individuals for genomic evaluation still face serious challenges. To begin with, raw NGS data are difficult to transfer even with high speed internet connection, and their bioinformatics’ analyses are expensive and require costly hardware equipment for the scale needed in animal or plant breeding. Often, wrong or incomplete reference genomes complicate the analyses; furthermore, a frequently overlooked issue of population studies using whole-genome sequence data is the high rate of missing SNP calls. This problem dramatically increases at low-depth sequencing. In addition, it should be noted that complete genome sequencing of whole breeding populations is unlikely in the near future, and thus, sequencing will be combined with other approaches such as array genotyping and/or genotype by sequencing. Currently, imputation is one of the most popular proposed solutions but, although accurate, imputation is not error-free either [4,7].

Unsurprisingly, assessing the advantages of NGS over array SNP genotypes for genomic selection is a debate. A fundamental issue is to predict whether complete or partial sequence data can improve upon existing SNP array-based selection strategies and, if so, in which cases. Here, to study the potential advantage of sequence- vs. array-based genomic selection, we adopted an optimistic view: we assumed that SNP genotypes obtained from NGS data were error-free, without missing data, and that as many individuals can be sequenced as genotyped and phenotyped. Of course these assumptions are quite unrealistic, and we attempted to model errors for some cases, even if imperfectly. Nevertheless, this initial error-free setting allowed us to study two important issues: to set up an upper bound on the limits of genomic selection and to fine-tune existing methods to improve their performance. Furthermore, given the increasing SNP density of current genotyping technologies and their allegedly low calling rates, the potential advantages of very high-density genotyping over NGS data or of genotyping by sequencing were explored. We also considered the effect of SNP ascertainment in the design of genotyping arrays.

## Methods

### General setting

We simulated SNP patterns via a mixed coalescence and gene-dropping approach, modeling a bottleneck that reflects domestication and modern breeding, while simultaneously conditioning on observed nucleotide diversity for cattle (nucleotide diversity π ~ 1.2 × 10^-3^ per nucleotide) [8]. First, we simulated 2N sequences, corresponding to N diploid individuals, using the coalescence method. A subset of those individuals was used to ascertain the SNPs on the array. Another, separate subset of individuals made up the founders in a complex pedigree, that is, individuals used to discover the array-SNPs were not in the pedigree. Genomes in the successive generations were obtained by gene-dropping. Molecular and phenotypic data were available for all individuals in the pedigree. Validation of the prediction was performed by removing the phenotypes of the individuals of the last generation; the correlation between true and estimated genetic merit was used as a measure of performance for each method (pedigree BLUP, low- and high-density arrays or sequence data), in the animal breeding literature, this correlation is commonly referred to as ‘accuracy’. Details of each step are in the following sections.

### Genome structure, genetic architecture and population history

We simulated a genome consisting of ten chromosomes, each 3 Mb long. This length corresponds to ~1% of a complete mammalian genome, which has a typical length of around 3 Gb. We assumed that causal mutations (quantitative trait nucleotides or QTN) could occur only within 65 ‘genes’, each 10 kb long, which corresponds to the average gene length in the human genome. The 65 genes were clustered on seven chromosomes, each chromosome harboring 20, 10, 10, 10, 5, 5 and 5 ‘genes’, respectively.

To simulate the data in the base population, we used the coalescence theory. For a general introduction to the coalescence, see e.g. Rosenberg and Nordborg [9]. In the coalescence simulations, we employed MaCS [10], running 10 iterates to mimic the 10 independent chromosomes. We used the same demographic model as in Pérez-Enciso [11], which comprises an ancient bottleneck and a current level of heterozygosity of 1.2 × 10^-3^ per site, which was the value reported for Holstein cattle by the bovine sequencing consortium [8], although it is slightly lower than that recently reported by Daetwyler et al. [4] (1.44 × 10^-3^). This latter work, nevertheless, refers to several breeds, among which some may present larger variability than that used here. Nevertheless, our results were insensitive to a reasonable range of nucleotide diversities (results not shown). In a limited number of cases, we also evaluated the effect of SNP ascertainment bias when SNPs were selected in a population different from the target population, as opposed to ascertaining the SNPs in the same population. To do this, we simulated data in a structured population to mimic two breeds, also using the same model as in Pérez-Enciso [11]. Coalescence commands are detailed in the Appendix.

Once base population sequences were simulated by the coalescence method, gene-dropping was performed without selection in a complex pedigree. The pedigree consisted of 4520 individuals and seven generations. In the base population, 20 sires were mated to 50 dams. In each successive generation, 20 sires were also mated to 50 dams, and each dam had two offspring with two sires, with a total of 500 individuals per generation. Mating was not completely hierarchical: as in real dairy cattle pedigrees, dams were mated to different sires to produce each offspring. Molecular and phenotypic information was assumed to be available for all individuals in the pedigree. Recombination rate was 1 cM/Mb, and no recombination interference was simulated, except that the maximum number of crossovers per chromosome per meiosis was set to 4.

To generate the phenotype of the i^th^ individual, a linear model including additive and dominant effects was used:

$$ {y}_i=\upmu +{\displaystyle {\sum}_q^{nQTN}\Big({\gamma}_{\mathrm{i}}{a}_{\mathrm{q}}}+{\delta}_{\mathrm{i}}{d}_{\mathrm{q}}\Big)+{e}_{\mathrm{i}}, $$

where *y* is the phenotype; μ is the general mean; nQTN is the number of QTN; γ is an indicator variable taking values -1, 0 and 1 for genotypes ‘00’, ‘01’, and ‘11’, respectively, 0 being the ancestral allele and 1 the derived allele; *a* is the additive substitution effect; δ is an indicator variable with value 1 for heterozygous genotypes and 0 otherwise; *d* is the dominant effect; and *e* is a normal residual *e* ~ N(0, Ve). The number of QTN fitted was either 20 or 100. QTN were randomly sampled among all SNPs positioned within the 65 genes, i.e., within 650 kb out of the 30 Mb simulated in total. Note that in our model there is no restriction on the number of QTN per ‘gene’: some genes may contain several QTN and some genes may not contain any and this varies from replicate to replicate. This aims at reflecting genetic heterogeneity, whereby different causal mutations in the same gene can all cause a shift in phenotypic mean.

**Figure 1:**
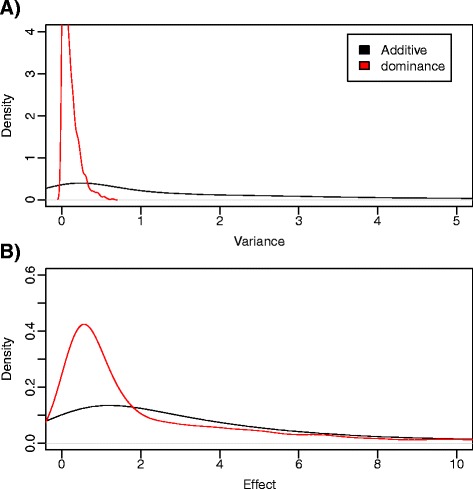
**Distribution of additive and dominant variances and genetic effects. (A)** Density of variances contributed by each QTN, both additive and dominant. **(B)** Density of genetic effects contributed by each QTN. Note the different scales in each graph.

As for the distribution of gene effects, *a* and *d*, most of the empirical evidence relates to the effects of new mutations [12,13], which do not necessarily follow the same distribution as that of the effects of segregating genes that underlie the complex traits under selection. Much less is known in this latter case than for new mutations. A uniform distribution of equal effects across genes is probably as unrealistic as is the assumption of a small number of major genes. A plausible scenario probably lies somewhere in between. An educated guess can be based on the review by Hayes and Goddard [14], who fitted a gamma distribution Γ(5.4,0.42) to reported locus substitution effects in dairy cattle. Here, we sampled the QTN additive variances (Va) – rather than the substitution effects – from a Γ(5.4,4.2), and dominance variances (Vd) from an exponential exp(10). Figure 1 shows these two distributions.

For each QTN, additive (*a*) and dominance (*d*) effects were then obtained from standard formulae [15]: Vd = [2 f (1-f) *d*]^2^ and Va = 2 f (1-f) [*a* + *d* (1-2f)]^2^, f being the frequency of the allele. This implies that low-frequency alleles explain *a priori* the same variance as intermediate-frequency alleles, which in turn implies that their absolute effects are larger. If this is the case, whole-genome sequence data will be more accurate than SNP-array genotypes, because the alleles in commercial SNP chips do not usually include low-frequency alleles [5]. Allele frequencies were calculated using the pedigree base population, that is, the 1020 individuals in the pedigree with unknown parents. Note that the same variance can be obtained with equal substitution effects of different signs, meaning that the same contribution to the variance can be assigned to a locus, irrespective of whether the derived allele has either a positive or a negative effect on the phenotype. Here, we sampled a positive or negative sign with equal probability. Note that this event is unlikely under a scenario of hard selective sweeps, for which only the derived allele is expected to be selected for, but it could reasonably occur under a model of selection on standing genetic variation, for which an allele can be segregating under a mutation-drift balance and then become rapidly selected for or against under artificial selection.

Finally, environmental variance (Ve) was adjusted such that the broad-sense heritability was *H*^*2*^ = 0.25. To do this, complete equilibrium was assumed, i.e.:

$$ {H}^2=\frac{{\displaystyle {\sum}_i^{nQTN}}\left(Vai+Vdi\right)}{{\displaystyle {\sum}_i^{nQTN}}\left(Vai+Vdi\right) + Ve}. $$

Alternatively, the actual additive and dominance variances, given the observed genotypes in each base population, can be computed and Ve can be adjusted exactly for the base population. In practice, in spite of disequilibrium, we observed only small differences between both strategies and thus we fitted Ve assuming equilibrium.

### Genetic evaluation and array-SNP ascertainment

We predicted genetic merit based on pedigree BLUP and on molecular information. For BLUP, we used broad heritability (H^2^ = 0.25) for evaluation instead of narrow sense heritability (h^2^), this had a negligible effect on results and the rationale is that true parameters are rarely known. Nevertheless, restricted maximum likelihood (REML) estimates of h^2^ in the simulated data were close to 0.25. For the molecular-based method, we considered the following ‘blind’ strategies:

1. A medium-density SNP array that comprised 7500 SNPs on average.
2. A high-density array that contained ~17 000 SNPs.
3. RAD (Restriction site associated DNA markers) sequencing: all SNPs in 100 subsets of the genome, each 10 kb long, were selected. This approach is intended to mimic genotype by sequencing (GBS) and, in our scenario, resulted in about 11 000 SNPs. This strategy could also be assimilated to exome sequencing that consists in sequencing a small subset of the genome i.e. the coding regions.
4. Sequence: all simulated SNPs in the population, ~335 000 SNPs.

For comparison, we also mimicked the use of biological information, which differed in its degree of accuracy (and inversely to plausibility):

1. Causal SNPs: all QTN, irrespective of their contribution to total variance, were included in the model (20 or 100 SNPs).
2. All SNPs within the defined ‘genes’, that is, the 65 10-kb regions where QTN were located ~7500 SNPs.
3. All SNPs within a subset of genes (50%) were included, to mimic incomplete biological information, about 3800 SNPs.
4. A subset of 50% of genes plus 30 ‘neutral’ 10-kb windows were included in the model, to mimic both incompleteness and incorrect biological information.

The first strategy would correspond to the maximum and unrealistically attainable accuracy. However, the latter three strategies cannot be deemed to be completely implausible: we are assuming that only the genes that contain the causal mutations are known, but not the causal mutations themselves. Note that this information should be available *a priori*. We shall come back to this issue later.

To simulate SNP ascertainment bias, as in commercial arrays, a subset of 50 diploid individuals (100 sequences) was selected among those simulated by the coalescence approach; SNPs that segregated in that subsample were chosen such that their minimum allele frequency (MAF) was at least 0.15 and hence randomly selected to mimic a 50 k and 700 k SNP density. In our simulations, this implied selecting ~0.9% and ~20%, respectively, of those SNPs with a MAF greater than 0.15. In the two-breed model, the SNPs were chosen from those segregating in the second breed. In a limited number of cases, we also evaluated the consequences of choosing a random set of SNPs, irrespective of frequency (MAF > 0). With whole-genome sequence data, all SNPs were included in the model irrespective of their frequency.

Genetic evaluation was based on the nonlinear A method of VanRaden [16]. Evaluation based on a genomic BLUP (GBLUP or RR-BLUP) model, also known as SNP-BLUP, gave very similar results that are not shown. In a limited number of cases, we also employed Vanraden’s nonlinear B method, which is comparable to Bayes B [17], but more efficient in computation time. The computation of the VanRaden's B method was, nevertheless, much slower than GBLUP or the A method. The model for the phenotype was:

1 $$ {y}_{\mathrm{i}} = \upmu + {\displaystyle {\sum}_j^{nSNP}\left({x}_{ij}-{\overline{x}}_j\right)\ {a}_j + {e}_{\mathrm{i}},} $$

where *x*_*ij*_ is the genotype, coded as -1, 0 and 1, and ${\overline{x}}_j$ is average genotype value (i.e., 1 minus twice the allelic frequency under Hardy-Weinberg equilibrium); and total genetic merit prediction was simply based on (1) and replacing *a*_*j*_ by their estimates:

2 $$ {\widehat{g}}_{\mathrm{i}} = \kern0.5em {\displaystyle {\sum}_j^{nSNP}\left({x}_{ij}-{\overline{x}}_j\right)\ {\widehat{a}}_j.} $$

Nonlinear A was solved using the solve-SNP program [18] that implements a preconditioned conjugate gradient algorithm, with outer loops for marker variance updates; the initial *a priori* variance for all SNP effects was ${\sigma}_{a0}^2={\displaystyle {\sum}_i^{nSNP}{\sigma}_{ai}^2/2}{\displaystyle {\sum}_i^{nSNP}{p}_i\left(1-{p}_i\right)}$, *p*_*i*_ being the allele frequency in the pedigree for the i^th^ SNP and *nSNP*, the number of SNPs. The variance of each SNP effect $\left({\sigma}_{ai}^2\right)$ is updated in each iteration as:

$$ {\sigma}_{ai}^2={\sigma}_{a0}^2\left({1.125}^{\frac{\left|{\widehat{a}}_i\right|}{sd\left({\widehat{a}}_1,\dots {\widehat{a}}_n\right)}-2}\right), $$

which in practice means that SNP effects that are smaller than two standard deviations are more shrunken, whereas SNP effects that are larger are less shrunken. Formal justification of this algorithm is in Gianola [19], who suggests a very similar iterative scheme to find the posterior mode of the well-known model BayesA. It should be noted that the term “variance of each SNP effect” has no meaning *per se,* because there is only one effect per locus; it can be seen as a computational device to find nonlinear estimates of SNP effects [19].

To compare the performance of the three strategies (i.e., pedigree BLUP, array genotyping, sequence-based), we computed the correlation between true and predicted genetic merit in a subset of individuals for which phenotypes had been removed (as a measure of predictive ability). For strategies using molecular information, Nonlinear A was used. In practice, the breeder is primarily interested in predicting the genetic merit of new individuals, and so cross-validation was performed by removing the phenotypes of the 500 last-generation individuals. One hundred replicates per case were run.

### Simulating sequencing and genotyping errors

For most results presented here, we assumed no missing data and no genotyping or sequencing errors. This is, as mentioned, a strong assumption. Modeling errors is, however, no easy task because they are neither unbiased nor independent in the case of NGS data. For NGS, sequencing depth is the most relevant factor but the bioinformatics pipeline and the population structure are also relevant [20]; furthermore, NGS genotyping errors are not random, and popular algorithms like samtools [21] or GATK [22] are biased towards the reference allele at low depth and at low frequency of the alternative allele [20]. For this reason, detecting rare variants can be difficult. In practice, imputation errors will also be present because, currently, no breeding program considers the sequencing of all selection candidates. In a previous study [11], we attempted a realistic simulation of all these sources of errors, but such an approach is extremely costly in terms of computer resources and is not feasible for the population sizes considered here.

Here, to mimic NGS errors efficiently (yet somewhat naively, we concede), we used a three-step procedure: (i) a biased genotyping error (λ model was applied (Table 1), (ii) SNPs with an allele count < K were removed, that is, we required an allele to be observed at least K times in order not to be taken as error, but we applied filtering after the NGS errors had occurred, since we assumed that this quality control is done after the genotypes have been called, and (iii) an imputation error γ in a subset of the individuals. Parameters in Table 1 reflect, qualitatively, the errors we found using our NGS simulator pipeline [23]. Base sequencing error rates λ = 0.05 and 0.01 were considered. For imputation, we considered α = 0.20 and 0.01 error rates, where rates were independent of genotype (Table 1). This range covers the most extreme bounds since an error rate as high as 20% was reported by Daetwyler et al. [4] for real cattle data, whereas 1% can be considered as the lowest attainable error [6]. We applied the imputation rate error to all individuals under the assumption that the number of fully sequenced individuals is likely to be very small compared to the total population size. Similarly, for array genotyping, we applied genotype independent error rates of 10^-3^ and 10^-4^ per genotype. This range spans most results from the literature [23-25].

**Table 1:** **NGS error rate and imputation error matrices**

**Real**	**Called (NGS)**			**Imputed**		
	**RR**	**RA**	**AA**	**RR**	**RA**	**AA**
RR	1 − λ/5	λ/5	0	1 − α	α/2	α/2
RA	λ	1 − 2λ	λ	α/2	1 − α	α/2
AA	0	λ/5	1 − λ/5	α/2	α/2	1 − α

R: reference allele; A: alternative allele; λ: base NGS genotyping error rate; α: imputation error rate. The Table shows the probability of a true genotype being called or imputed for each of the three possible genotypes; for instance, under this model, a true heterozygous genotype RA has a probability λ of being called as homozygous RR or AA and 2λ of being wrongly called.

## Results

Figure 1 shows the distribution of variances, f(Va) and f(Vd) and of absolute gene effects as predicted by our model. Figure 1A shows the distribution of additive and dominant variances, that is, Va ~ Γ(5.4,0.42) and Vd ~ exp(10). The distribution of gene effects was obtained by integrating f(Va) and f(Vd) over the full spectrum of site frequency (Figure 1B) and therefore the variances of the gene effects are larger (i.e., flatter distributions are observed) than those of Va and Vd. Note that we assumed independence of gene effect and frequency, which is unlikely for a trait under strong selection but may be a reasonable approximation with a large number of loci. However, in the absence of detailed empirical evidence, our model captures the plausible situation for which a few genes may have a large effect but most of the variance is explained by a larger number of loci with a small effect [26,27,28]. Note that contributions of each locus to genetic variance are dynamic, since they depend on allele frequencies that evolve under artificial selection.

**Figure 2:**
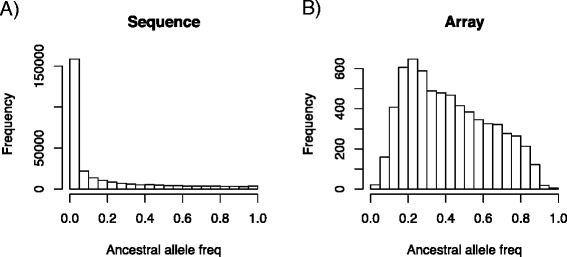
**Unfolded site frequency spectra.** Site frequency spectra found for complete sequence **(A)** and for the high density array **(B)** when SNPs are ascertained in a panel of 50 individuals from the same population, setting a MAF > 0.15. Data shown correspond to data from the whole pedigree.

For the simulated sequence of 30 Mb, an average of 335 000 SNPs were obtained from the coalescence simulations. The unfolded site frequency spectrum (SFS) for individuals in the pedigree is in Figure 2A. Note that most variants have a low frequency (f < 0.05), which is a logical outcome of the coalescence process. A fundamental difference between SNP and sequence genotypes is that the former data are affected by ascertainment bias (Figure 2B). Indeed, it shows the well-known highly distorted site frequency spectrum due to ascertainment bias, for which SNPs with high MAF are over-represented. Therefore, the way SNPs are chosen can lead to differences between genomic selection strategies [5].

To study ascertainment effect on prediction accuracy, scenarios that differ in the choice of MAF and ascertainment in one or more breeds were compared (Table 2). The first interesting observation is that setting a moderate minimum MAF (>0.15) for the SNP discovery panel improved prediction accuracy more than not filtering the SNPs (MAF > 0), which is explained by the fact that most variants are rare variants (Figure 2B); indeed, setting a minimum MAF when ascertaining SNPs increases the probability that the markers segregate in the whole population and, as a result, increases the probability that they are informative in the cross-validation panel. Discovering the SNPs in a breed different to that under study also decreases prediction accuracy. Even at very low differentiation levels (Fst ~0.05), using ascertained SNPs in another breed leads to decreased prediction accuracy. Nevertheless, the effect of ascertainment seems to be modest and tends to decrease with increasing SNP density. In the following, we present only the results that were obtained when SNP ascertainment was done in the same breed as that under study and only SNPs with a MAF greater than 0.15 were included.

**Table 2:** **Effect of SNP ascertainment scenario on prediction accuracy**

**Array size (Nb of SNPs)**	**Ascertainment breed**	**MAF**	**Prediction accuracy (SD)**
Medium-density (7 k)	Same	0.00	0.41 (0.07)
	Same	0.15	0.45 (0.08)
	Different	0.15	0.40 (0.08)
High-density (17 k)	Same	0.00	0.44 (0.08)
	Same	0.15	0.47 (0.08)
	Different	0.15	0.43 (0.08)

The medium-density array contains ~ 7.5 k SNPs; the high-density array contains ~17 k SNPs; the trait is affected by 100 QTN and h^2^ = 0.25; results are averages of 100 replicates.

**Figure 3:**
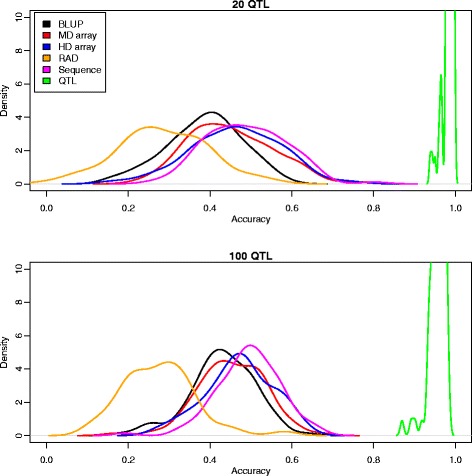
**Distribution of accuracies across replicates with different strategies.**

Table 3 and Figure 3 summarize the main results of the comparison of the performance between methods (pedigree BLUP, array genotyping, RAD, whole-genome sequence and causal SNPs) and for two genetic architectures, namely 20 or 100 QTN. In all cases, the number of genes that potentially carry QTN was set to 65 and they were distributed among seven of the 10 chromosomes (see Methods section). As is well known, genomic selection can improve upon pedigree BLUP; in our simulation scenario, the increase in prediction accuracy was ~10 to 20% when using the high-density SNP array (Table 3). Using the sequence data, there was a 4% improvement in accuracy compared to the HD array. An important result of Table 3 is the evident law of diminishing returns with SNP density: as the number of SNPs increased, the differences between sequence and SNP genotyping tended to disappear. This is perhaps clearer in Figure 3. Overall, this is a consequence of most SNPs being redundant because of linkage disequilibrium and because they are not very informative, since most variants are rare (Figure 2). Nevertheless, how these SNPs are chosen is important. For RAD-sequencing, prediction accuracy was actually much lower than for the medium density array, despite the fact that 45% more SNPs were used. The likely reason is twofold: with RAD-sequencing, many SNPs are tightly linked whereas SNPs in arrays are uniformly distributed along the genome and second, SNPs in arrays tend to be more informative than those obtained by direct sequencing due to MAF restrictions. In the limited number of cases tested, the nonlinear B method resulted in comparable accuracies as for nonlinear A, and we did not observe a large gain when using sequence vs. the HD array (Table 4).

**Table 3:** **Accuracy obtained with different strategies**

**Method**	Number of SNPs*	**Number of QTN**	
		**20**	**100**
Pedigree BLUP	-	0.38 (0.09)	0.43 (0.09)
RAD	11,000	0.28 (0.10)	0.27 (0.09)
Medium-density array	7,500	0.45 (0.09)	0.45 (0.09)
High-density array	17,000	0.47 (0.08)	0.47 (0.08)
Sequence	335,000	0.49 (0.10)	0.49 (0.08)
Causal SNPs	20/100	0.98 (0.01)	0.95 (0.02)

Accuracy is measured as the correlation between true and predicted breeding values of the last generation individuals in cross-validation and is the average of 100 replicates; *number of SNPs used for prediction; for Sequence, all SNPs are used; for causal SNPs, only 20 or 100 QTN are used.

**Table 4:** **Accuracy obtained using the non linearB method**

**Method**	Number of SNPs*	**Number of QTN**	
		**20**	**100**
High-density array	17 000	0.52 (0.19)	0.46 (0.08)
Sequence	335 000	0.49 (0.11)	0.46 (0.11)
Causal SNPs	20/100	0.98 (0.01)	0.95 (0.01)

Accuracy is measured as the correlation between true and predicted breeding values of last generation individuals in cross-validation and is the average of 50 replicates; *number of SNPs used for prediction; for Sequence, all SNPs are used, for causal SNPs, only 20 or 100 QTN are used.

The second important conclusion from Table 3 and Figure 3 is that there is still much room for improvement, i.e., when causal SNPs (the QTN) were known, prediction accuracy was greater than 95%. In this case, a mild effect of the number of QTN was observed. Given that all QTN are within the whole set of SNPs, it is evident that the performance of sequence-based methods can be dramatically boosted by carefully assigning biologically meaningful priors. Certainly, knowing the QTN is an extreme, unrealistic scenario of prior information, whereby all non-causal SNPs are removed from the model and the only uncertainty lies in the estimation of the effect of each causal mutation.

**Figure 4:**
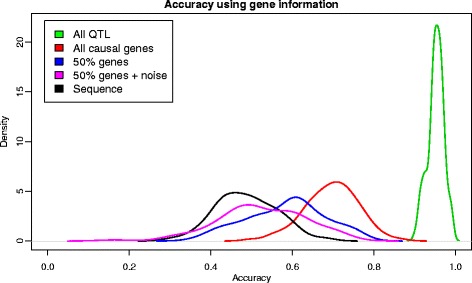
**Use of biological prior information.** Lines corresponds to accuracy using all QTN in the model (green), all SNPs within the 65 genes (red), all SNPs in 50% of the genes (blue), or SNPs in 50% of the genes and 30 random windows (magenta); in black, when all SNPs are included in the model. The number of QTN is 100.

We investigated milder and more realistic options than knowing all QTN (see Methods). The next most favorable situation would be to know all the genes that might contain the causal variants. This resulted in a model containing ~7000 SNPs on average. Importantly, this strategy resulted in prediction accuracy equal to 0.69, which was 40% higher than with the sequence-based strategy (Figure 4). Since biological knowledge is always limited, we also considered incomplete or partially incorrect specified models. Including only a subset (50%) of all causative genes in the model or a 50% subset of causal genes with 30 additional neutral windows resulted in poorer accuracy predictions that were similar to those obtained by using whole-genome sequence data (Figure 3). In these cases, average accuracies were equal to 0.52 and 0.55 when 30 random windows were included or not in the model, respectively. Although the evidence is limited, these results suggest that the performance of the predictor is quite sensitive to accurate and complete prior information.

**Figure 5:**
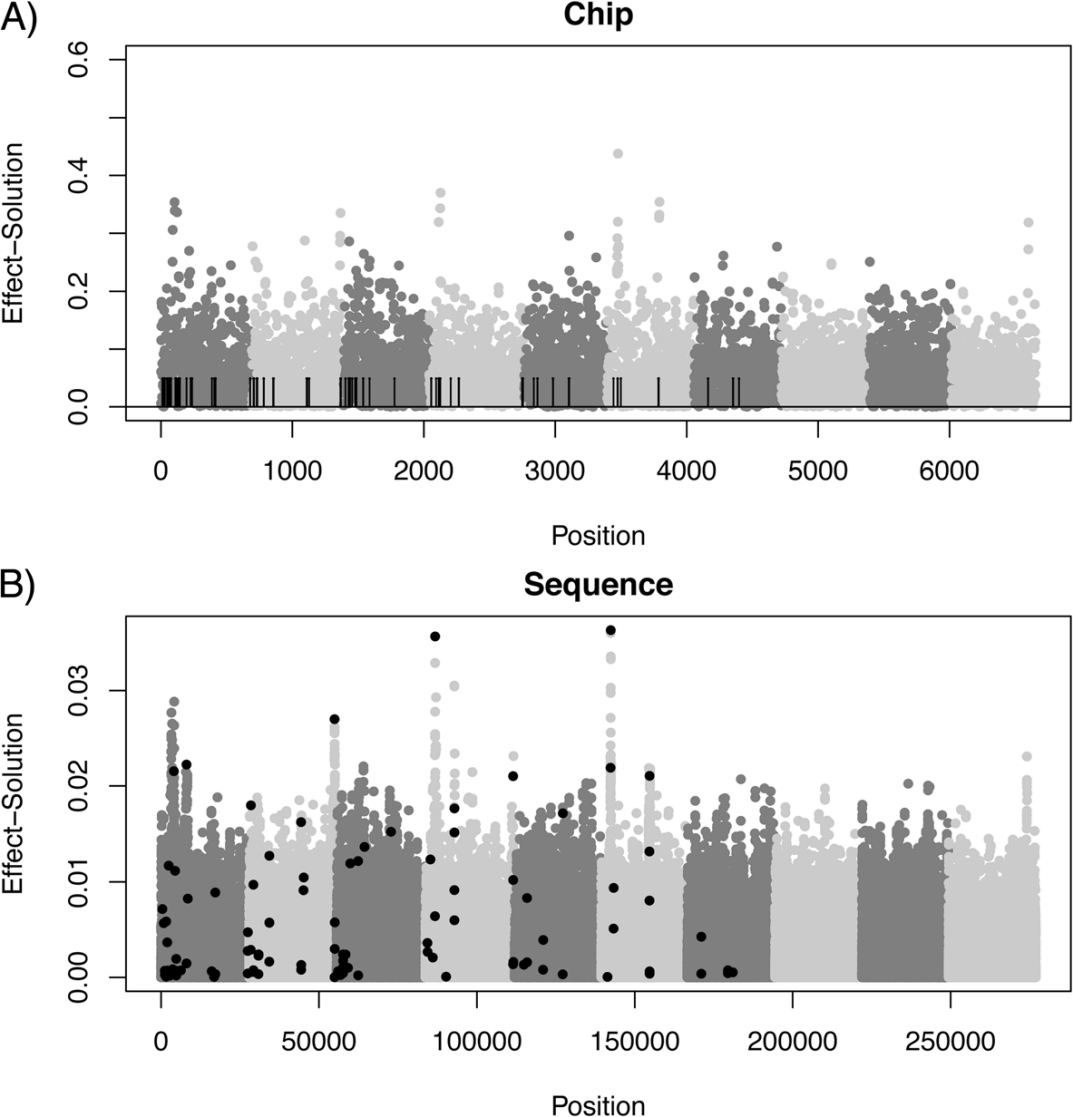
**Manhattan plot of SNP effect estimates. A)** high-density chip, dashes represent causal loci positions; **B)** sequence results, black dots are the causal loci. Each chromosome is represented in a different shade of grey; the last three chromosomes do not contain any QTN. Effects are absolute values.

**Figure 6:**
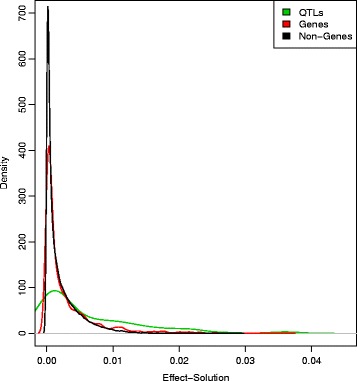
**Distributions of SNP effect estimates.** Distributions corresponding to intergenic SNPs (black), SNPs within genes (red) and QTN (green) obtained from sequence data analysis.

In all the predictors considered here, equal priors were assigned to each SNP, and our simulations suggest that the increase in information with sequence data is offset by added noise in the prediction compared to HD genotyping. This is illustrated in Figure 5, which presents the Manhattan plots of SNP effects on a random replicate for a medium-density chip (Figure 5A) and whole-genome sequence data (Figure 5B). Even with whole-genome sequence data, larger weights in the predictor (Equation 2) were not necessarily assigned to the causal SNPs (black dots) compared to neutral SNPs, although the largest effects do tend to be picked up. Moreover, the profile of the last three chromosomes (i.e., those that did not carry any QTN) was not very different from that of the other chromosomes that did contain QTN: for example, compare chromosomes 7 and 8. Unsurprisingly, the panorama is rather blurred when SNP arrays instead of whole-genome sequence data are considered (Figure 5A vs. 5B). Note that the scales of the two graphs in Figure 5 are quite different: SNP effect estimates are much more shrunken towards 0 with whole-genome sequence data than with array data. Figure 6 shows the distribution of SNP effects obtained with a sequence-based analysis according to whether they are causal, located within genes, or elsewhere.

**Figure 7:**
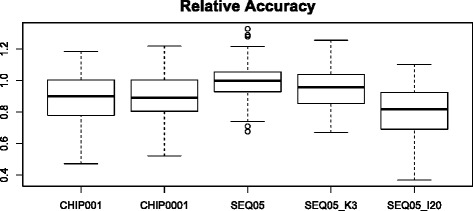
**Accuracy with several error models.** All data refer to a 100 QTN model and are the average of 100 replicates, chip refers to the high-density array. CHIP001: 10^-3^ genotyping error; CHIP0001: 10^-4^ genotyping error; SEQ05: sequence error λ = 0.05, imputation error γ = 0.001, minimum K number for an allele to be considered K = 1 (all SNPs are considered); SEQ05_K3, as previous model with K = 3; SEQ05_I20: as previous model with γ = 0.20. Table 1 describes the error models. Values represented are relative to accuracy obtained with full sequence without errors.

Prediction accuracies obtained with the different sequencing or genotype error models are in Figure 7. Overall, the effects were small but several interesting points should be noted. First, the current error rate of SNP genotyping technologies (10^-3^ to 10^-4^) is unlikely to have a strong influence on prediction accuracy. Given that the loss in prediction accuracy observed with the HD chip compared to whole-genome sequence data is ~4% (Table 3), genotyping errors may cause an additional loss of 4%, or 0.45 vs. 0.47. As for sequence-based methods, the impact of errors was negligible, unless the imputation error rate increased to about 20%. Note that the recent sequencing of 234 bulls [4] reported an imputation accuracy of 80%. At such high imputation error rates, the gain in accuracy of sequence over array genotypes is not guaranteed. Removing very rare variants (K = 3) did not change the prediction accuracy.

## Discussion

The use of whole-genome sequence data for the prediction of genetic merit creates major challenges, primarily in terms of bioinformatics and computational demands, but also from a statistical point of view. Notwithstanding, given that the sequencing prices still continue to decrease, this topic is attracting much interest and is a highly active area in the field of quantitative genetics. To date, the few published investigations do not allow us to draw absolute conclusions. Thus, the advantage of whole-genome sequence data over very high-density genotyping is still unclear, even with total absence of sequencing errors and of missing data (which is equivalent to an error-free imputation process). Overall, our simulations suggest that the availability of whole-genome sequence data could increase prediction accuracy, measured as correlation between predicted and true genetic value in the last generation, by 4 to 8% compared to SNP arrays. This increase is much lower than the results reported by Meuwissen and Goddard [3], who suggested that increases of 40% could be attained, although in a population with low disequilibrium. Other authors i.e., Druet et al. [5] reported that the advantage depended on the genetic architecture; the largest increase was observed when all causal mutations were rare. Meuwissen and Goddard [3] reported that a nonlinear predictor such as BayesB gave much higher accuracies than a linear predictor (RR-BLUP), whereas Druet et al. [5] did not use RR-BLUP. However, in our case, both the linear RR-BLUP and the nonlinear A (comparable to BayesA) gave similar results, RR-BLUP resulting in accuracy reductions of ~0.02% across scenarios (results not shown). As for nonlinear-B, we did not find either an improvement when using sequence (Table 4). Nonlinear A and nonlinear B are predictors that can pick up very large QTN effects, with estimates as large as 0.5 genetic standard deviations for a single marker, which makes it comparable to the nonlinear predictors of the other authors [29]. More recently, Macleod et al. [30] showed by simulation that low linkage disequilibrium has a strong effect in increasing the advantage of sequence over high density genotyping. Given that rather different genetic and simulation scenarios were used in all these studies, it is somewhat difficult to compare them.

Interestingly, Hayes et al. [31] recently reported a minimal increase (0 to 3% depending on trait) when sequencing followed by imputation vs. SNP array genotyping were compared on real dairy cattle data. Our work suggests at least three reasons to explain this result. First, there is a large uncertainty on the outcome of the selection and drift processes involved, a natural consequence of the coalescence process that directly affects disequilibrium, and hence predictive ability. Figure 3 clearly shows this variability, which corresponds to a 30 Mb sequence, divided in 10 independent chromosomes. Although variability along a whole mammalian genome would be smaller, it will certainly exist. Second, most SNPs are rare and in strong disequilibrium. This means that they will be difficult to pick up in any cross-validation study (that is, most likely rare alleles will not be sampled in both the reference and target populations simultaneously), irrespective of whether they contribute to variance or not. The third reason is imputation errors rather than sequencing errors. Most imputation errors occur for low-frequency variants, but these are the most frequent variants. As a result, added noise can hamper the potential advantages of sequence-based methods (Figure 7).

Furthermore, our results also show that prediction accuracy plateaus as SNP density increases. This phenomenon was already reported by Ober et al. [32], who did not observe any clear change in accuracy when using either 2.5 million or 150 k SNPs for an analysis on starvation stress in Drosophila, although in a very small population (157 lines). Vanraden et al. [6,33] also observed that gain in accuracy declined gradually when passing from 10 k, 20 k and 40 k to high-density (more than 700 k) SNP chips in dairy cattle data. In our simulated scenario, prediction accuracies obtained with whole-genome sequence data were close to those attained with high-density chips containing 17 k SNPs. Although the SNP density at which accuracy plateaus will vary depending on the specific scenario and on the genetic architecture of the trait, there is little doubt that a small percentage of all SNPs that segregate in the population may be sufficient to make predictions as accurate as with whole-genome sequence data. The question is then whether it will be cheaper to sequence at very sparse coverage and impute sequence variants to SNP genotypes obtained with very high-density arrays. Genotyping by sequencing has been proposed as an interesting alternative to high-density genotyping [34]. Although we have not compared all these strategies in detail here, our results suggest that genotyping by sequencing will not pay off because it suffers from the same limitations as sequencing, i.e., the use of an excess of tightly linked and lowly informative markers. As for sparse sequencing and imputation, the success of this strategy may depend critically on error rates. In a recent work [11], we compared how much molecular relationship matrices computed using either complete sequence or ascertained SNPs were similar. We concluded that, at least with relatively simple demographic scenarios (without admixture), the correlation was as high with NGS data as with HD SNP array data. Thus, we suggested that producing complete sequences was probably unnecessary, and simulations presented here tend to support this hypothesis, provided no external, biologically meaningful information is used.

In fact, we have shown that that there is still ample room for improvement, as evident from the large increase in prediction accuracy that could be obtained if QTN were known. In practice, decades of research have shown that this is highly unlikely to happen, and many causal variants will remain undiscovered even in the largest GWAS (genome-wide association studies) experiments [35] as shown in Figures 5 and 6. Using the most significant SNPs may not increase prediction either. In our study, we found that the distributions of SNP effects (causal, genic and intergenic) overlap (Figure 6), which suggests that including or not a SNP in a model based on its estimated effect may not have a strong impact.

A much less ambitious target is the characterization of genomic regions that are involved in the determination of a given phenotype, and here we have shown that this can result in a dramatic improvement of about 40% (in our scenario) compared to the use of whole-genome sequence data. This suggests that one main reason why whole-genome sequencing does not meet expectations – compared to high-density genotyping – is ‘simply’ the use of wrong prior information. In all the predictors considered here, equal priors are assigned to each SNP (this is true also for Bayesian regressions such as the Lasso or BayesB), and our simulations suggest that the increase in information provided by whole-genome sequence data is offset by added noise in the prediction.

An unfortunate side effect is that even moderate errors when determining the set of true QTN can reduce this advantage (Figure 4). For future studies, the important message is that incorporating meaningful biological knowledge into the predictor can make a difference, and that research to uncover causative genes and or polymorphisms is well justified. At this point, much research remains to be done on optimum approaches for assigning priors using biological information, but there are several ongoing projects in this area. Macleod et al. [36] and Hayes et al. [31] have suggested to partition the mutations according to functional classes, combining it with BayesR. Ideally, approaches that combine functional information (say gene ontologies or presence of certain sequence motifs like transcription factors) with empirical information from population genetic studies (say selective sweeps evidence, although see Kemper et al. [37] for how this can be misleading) could be considered. In this setting, it may make sense to obtain a whole-genome sequence and accurately assign priors to each set of polymorphisms. Alternatively, specialized arrays with an optimum set of SNPs could be designed.

**Figure 8:**
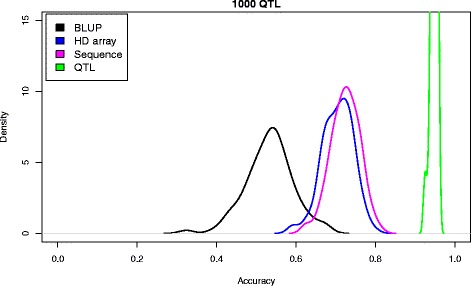
**Accuracy across strategies with a near infinitesimal model.** The results correspond to a 1000 QTN model with additive equal effects and no dominance.

Without doubt, genetic architecture affects the performance of genomic selection, with and without the use of whole-genome sequence data. Unfortunately, there is much uncertainty on the precise genetic architecture of quantitative traits under selection. Therefore, all simulation studies, including ours, should be taken with some caution. Two extreme architectures have been traditionally considered: (1) a major gene model in which a few genes explain most of the genetic variance and (2) the infinitesimal model. The former does not explain the wealth of experimental data that has accumulated along the last decades whereas the latter, while not being biologically sound, can be considered as operationally correct for prediction purposes. Here, we chose an intermediate architecture that comprises a reasonable large number of loci, but each with a different effect. To study the potential effects of increasing the number of loci, even if briefly, we also simulated a model with 1000 loci that all have an equal effect and are randomly distributed among 10 chromosomes. The results (Figure 8) were not completely unexpected since they showed an increased relative advantage of all methods, and a smaller associated variance, compared to previous results (Figure 3). Importantly, the ranking of the methods was the same as for the oligogenic models used here; in particular, it is worth noting the large advantage that could still be obtained if the 1000 causal mutations were known. Therefore, in general, it seems that our results (Table 3, Figure 3) are robust to the underlying genetic architecture, although they suggest that the relative advantage of sequence data decreases when the number of causal loci increases.

A persistent matter of concern in genomic data is the occurrence of errors, due to the difficulty of ensuring uniform quality control in such massive datasets. For most of the simulations investigated in this work, we assumed that errors and missing data were absent and this is, admittedly, a strong assumption. In this sense, our results should be interpreted as the uppermost attainable limits, for the genetic architecture and experimental designs considered. Nevertheless, we also evaluated in a simplified manner the potential impact of sequencing and genotyping errors and we found (Figure 7) that, unless imputation errors are really high, they should not affect the results strongly. In practice, imputation errors can be high for low-frequency variants, as reported by Daetwyler et al. [4]. Note that the recent sequencing of 234 bulls [4] reported an imputation accuracy of 80%, which is rather low compared to the typical values greater than 95% reported with 6k to high-density SNP arrays [6]. With genotyping, a small decrease in prediction accuracy is expected for the typical error rates that occur with standard platforms (10^-3^ to 10^-4^). It is also expected that, with the ongoing development of improved technology and imputation tools, errors will tend to decrease.

The issue of SNP ascertainment bias in the design of arrays has received considerable interest, in particular for its effects on the inference of genetic parameters [38]. The main effect of ascertainment bias is that it modifies the SFS (Figure 2), and therefore genetic inferences based on the SFS will be difficult to interpret. However, the effects of ascertainment on genomic selection have been much less studied. Here, we found that ascertainment bias can have a moderate effect on prediction accuracy but the effect decreases as SNP density increases. Importantly, we found that setting a minimum MAF requirement had a beneficial effect on prediction, because it increases the informativity of the markers. In summary, our study suggests that the ascertainment process may not have a strong impact on accuracy of genomic selection provided SNP density is high enough. This conclusion holds at least for simple demographic scenarios such as those investigated here. In scenarios with more complex histories that involve admixture between distant lineages, such as for pig, ascertainment may have a stronger influence than in dairy cattle [11].

Here, we have only considered the case of single-breed evaluation. The more distant, genetically, the breed of interest is from the breed(s) used to ascertain the SNPs in the array, the worse is the array expected to perform compared to direct sequencing [11]. Prediction across multiple breeds should benefit from NGS data due to an increased persistence in accuracy over generations, compared to array-based predictions [3]. Nevertheless, the impact of NGS for this application needs further investigation.

## Conclusions

The availability of whole-genome sequence data can lead to increased selection response compared to current strategies based on medium-density SNP arrays but this increase*, per se*, is likely to be minimal, at least in a single-breed scenario. This conclusion seems relatively insensitive to the SNP ascertainment process, number of causal loci, presence of dominance and to modest genotyping or sequencing errors. In contrast, our work shows that a dramatic increase in prediction accuracy can be attained by using correct prior information. However, and importantly, this advantage can be quickly removed if the biological information used is partially incorrect or incomplete. Therefore, an improved biological understanding of the trait(s) is critical. However, in commercial breeding schemes selection targets are continuously changing to adapt to market requirements. This is equivalent to continuously modifying, even if partially, the genetic basis of the trait(s) under selection, which hinders the use of biological prior information. Furthermore, as shown, e.g., by the ENCODE project, many functional motifs in the genome are outside the coding regions, which may make it difficult to precisely delineate potential causal regions for any given trait. For all these reasons, we recommend caution on this issue that also requires further methodological developments.

Along this vein, although our results were relatively robust to genetic architecture, it is necessary to better understand and ascertain the genetic basis of complex traits. This implies characterizing not only the distribution of the effects of causative alleles and their frequencies, but also the influence of complex demographic scenarios that may include admixture events and multiple bottlenecks, quite frequent in livestock and plant species. For instance, Lohmueller [39] has predicted an excess of deleterious SNPs as a consequence of the exponential growth of the population in humans. Although exponential growth is unlikely to have occurred in livestock, the important issue is that demography can affect the genetic architecture of traits and, indirectly, the performance of genomic selection. Finally, despite widespread evidence of additivity [40], the effect of epistasis also merits further research. Overall, a better characterization of the genetic architecture of the traits under selection should help to successfully include biological information into genomic selection procedures. This is an active area of research and many important topics remain to be investigated, e.g., a better characterization of the genetic architecture of the traits under selection, methods to successfully include biological information into genomic selection procedures or the usefulness of sequence in multi-breed populations.

## Appendix

### Coalescence commands

The single-population coalescence command was:

macs N 3e6 -i 10 -t 0.0012 -r 0.001 -eN 1.67 10,

where N is the total number of sequences (i.e., twice the number of individuals), -i specifies the number of chromosomes (10) of length 3 Mb, -t specifies the nucleotide diversity per base pair, -r, the scaled recombination rate (in 4Ne units), and -eN specifies the bottleneck parameters. Properties of this model and its expected genomic relationship matrix are in Pérez-Enciso [11]. The command to simulate the structured population was:

macs N 3e6 –i 10 -t 0.0012 -r 0.001 -I 2 N1 100

-ej 0.042 2 1 -eN 1.67 10,

where N1 is the number of founder sequences in breed 1, and 100 is the number of sequences sampled in breed 2, where SNPs are ascertained, N = N1 + 100, -ej specifies time of splitting between the two breeds. This model results in mild structuring (Fst ~0.05).
